# Anabolic and Antiresorptive Osteoporosis Treatment: Trends, Costs, and Sequence in a Commercially Insured Population, 2003–2021

**DOI:** 10.1002/jbm4.10800

**Published:** 2023-07-24

**Authors:** Harsh Wadhwa, Janet Y Wu, Jennifer S Lee, Corinna C Zygourakis

**Affiliations:** ^1^ School of Medicine Stanford University Stanford CA USA; ^2^ Division of Endocrinology, Gerontology, and Metabolism, Department of Medicine Stanford University Medical Center Stanford CA USA; ^3^ Department of Neurosurgery Stanford University Medical Center Stanford CA USA

**Keywords:** ANABOLIC, ANTIRESORPTIVE, OSTEOPOROSIS, OUT‐OF‐POCKET COST, TRENDS

## Abstract

New anabolic medications (abaloparatide and romosozumab) were recently approved for osteoporosis, and data suggest that prescribing antiresorptive medications after a course of anabolic medications offers better outcomes. This study aimed to characterize prescription trends, demographics, geographical distributions, out‐of‐pocket costs, and treatment sequences for anabolic and antiresorptive osteoporosis medications. Using a commercial claims database (Clinformatics Data Mart), adult patients with osteoporosis from 2003 to 2021 were retrospectively reviewed and stratified based on osteoporosis medication class. Patient demographics and socioeconomic variables, provider types, and out‐of‐pocket costs were collected. Multivariable regression analyses were used to identify independent predictors of receiving osteoporosis treatment. A total of 2,988,826 patients with osteoporosis were identified; 616,635 (20.6%) received treatment. Patients who were female, Hispanic or Asian, in the Western US, had higher net worth, or had greater comorbidity burden were more likely to receive osteoporosis medications. Among patients who received medication, 31,112 (5.0%) received anabolic medication; these were more likely to be younger, White patients with higher education level, net worth, and greater comorbidity burden. Providers who prescribed the most anabolic medications were rheumatologists (18.5%), endocrinologists (16.8%), and general internists (15.3%). Osteoporosis medication prescriptions increased fourfold from 2003 to 2020, whereas anabolic medication prescriptions did not increase at this rate. Median out‐of‐pocket costs were $17 higher for anabolic than antiresorptive medications, though costs for anabolic medications decreased significantly from 2003 to 2020 (compound annual growth rate: −0.6%). A total of 8388 (1.4%) patients tried two or more osteoporosis medications, and 0.6% followed the optimal treatment sequence. Prescription of anabolic osteoporosis medications has not kept pace with overall osteoporosis treatment, and there are socioeconomic disparities in anabolic medication prescription, potentially driven by higher median out‐of‐pocket costs. Although prescribing antiresorptive medications after a course of anabolic medications offers better outcomes, this treatment sequence occurred in only 0.6% of the study cohort. © 2023 The Authors. *JBMR Plus* published by Wiley Periodicals LLC on behalf of American Society for Bone and Mineral Research.

## Graphical abstract

Geography of anabolic osteoporosis medication prescription is variable.

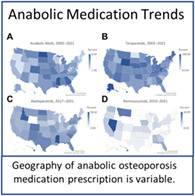

## Introduction

Osteoporosis is characterized by poor bone mineral density (BMD) that predisposes patients to fragility fractures and associated complications.^(^ ^1^ ^)^ Osteoporosis diagnoses are more common in women because of lower peak bone mass, longer life expectancy, and postmenopausal decreases in estrogen, which helps preserve bone mass.^(^ ^1^ ^)^ Though prevalence of osteoporosis is higher among women, mortality due to osteoporotic hip and vertebral fractures is higher among men.^(^ ^2^, ^3^, ^4^ ^)^ Increased life expectancies and aging populations worldwide have greatly increased the global burden of low BMD‐related complications; disability‐adjusted life‐years (DALYs) and deaths have doubled from 1990 to 2019.^(^ ^5^ ^)^

Treatment options for osteoporosis include two broad categories: antiresorptive and anabolic medications. Antiresorptive medications work to primarily decrease bone resorption by osteoclasts and include bisphosphonates and denosumab, as well as second‐line treatments like selective estrogen receptor modulators and calcitonin.^(^ ^6^, ^7^, ^8^ ^)^ Anabolic medications stimulate bone growth by directly or indirectly increasing osteoblast activity and include teriparatide, abaloparatide, and romosozumab.^(^ ^9^ ^)^ Antiresorptive medications are generally older—the first anabolic agent, teriparatide, was approved by the US Food and Drug Administration (FDA) in 2002, whereas abaloparatide and romosozumab were more recently approved in 2017 and 2019, respectively. Bisphosphonates are commonly prescribed as first‐line treatment for osteoporosis patients.^(^ ^10^ ^)^ However, there is growing evidence that anabolic medications are more effective at decreasing fracture risk and increasing BMD than antiresorptive medications and that the more common prescription sequence of antiresorptive therapy followed by anabolic therapy may not be the most effective.^(^ ^10^, ^11^, ^12^ ^)^

With the development of new anabolic agents and evolving treatment guidelines, understanding prescription trends of antiresorptive and anabolic medications for osteoporosis patients is crucial to improving osteoporosis care. Our primary aim was to study prescription trends of anabolic osteoporosis medications from 2003 to 2021. We hypothesized that anabolic medication prescription would increase over the study period, relative to antiresorptive medication prescription. Our secondary aims involved further characterizing patient demographics, geographical distributions, out‐of‐pocket costs, and treatment sequences for different osteoporosis medications.

## Materials and Methods

This study used a 100% sample of Optum's deidentified Clinformatics Data Mart (CDM) Database, which includes data on commercially insured and Medicare Advantage beneficiaries from January 2003 to March 2021.^(^ ^13^ ^)^ This study was approved by the Stanford University Institutional Review Board. All data used were deidentified and thus informed consent was not needed.

International Classification of Diseases, Ninth Revision, Clinical Modification (ICD‐9‐CM) and International Classification of Diseases, Tenth Revision, Clinical Modification (ICD‐10‐CM) codes were used to identify adult patients diagnosed with osteoporosis from 2003 to 2021. National Drug Code (NDC) identifiers were then used to stratify this cohort by whether they were treated with any osteoporosis medication (Supplemental Table S1). Patients were stratified further by treatment with anabolic (teriparatide, abaloparatide, or romosozumab) versus antiresorptive (alendronate, calcitonin, denosumab, etidronate, ibandronate, pamidronate, raloxifene, risedronate, or zoledronate) osteoporosis medications. Patients under age 18 years were excluded. Demographics and baseline characteristics, including age, sex, region of the United States (US), and Charlson Comorbidity Index (CCI) were collected. CCI was calculated using ICD‐9 and ICD‐10 codes as previously described.^(^ ^14^, ^15^ ^)^ Socioeconomic variables such as race, education level, and net worth were also collected.

The primary outcome of this study was the trend in prescription of anabolic osteoporosis medications over the study period. We tested the null hypothesis that there was no difference in rates of new prescriptions of anabolic osteoporosis medications from 2003 to 2021. Secondary outcomes included geographic distribution of anabolic medication prescription, distribution of anabolic prescription provider type, out‐of‐pocket (OOP) costs, and osteoporosis treatment sequence. Out‐of‐pocket costs were calculated as the sum of deductible, copay, and coinsurance. Costs were adjusted for inflation using the US Consumer Price Index rates and expressed in December 2021 US dollars.

### Statistical analysis

An a priori power analysis for linear regression trends demonstrated that 3134 patients are needed to provide 80% power for detecting a 5% change in the amount of anabolic medication prescriptions (α = 0.05). Trends were analyzed via a Cochran‐Armitage test. Kruskal–Wallis and chi‐square test were utilized to compare continuous and categorical variables, respectively. Multivariable logistic regression was used to identify predictors of receiving osteoporosis treatment and receiving anabolic medication. The change in medication out‐of‐pocket cost over the study period was calculated using compound annual growth rate (CAGR) as previously described.^(^ ^16^ ^)^ Since only partial data were available in CDM for 2021, trend projections for a full year of data were calculated. Projected out‐of‐pocket costs could not be calculated because of the variable nature of the data. Length of time spent taking medications was calculated by the difference in dates between the first and last prescription fills, then adding the number of days of the last refill. Diagrams were generated to visually represent sequence of osteoporosis medications trialed by each unique patient. The significance level was defined as a two‐sided α < 0.05. All statistical analyses were performed using SAS Enterprise Guide (SAS Institute, Cary, NC, USA).

## Results

In total, 2,988,826 patients with osteoporosis were identified. Of these, 616,635 patients (20.6%) were prescribed medication for osteoporosis. Patients who were female, Hispanic or Asian, from the Western US, had higher net worth, or had greater comorbidity burden were more likely to receive osteoporosis medication (Supplemental Table S2). Upon multivariable logistic regression, all of these variables were independent predictors of receiving osteoporosis medication (Table 1).

**Table 1:** Multivariable Logistic Regression of Patients With Osteoporosis Treated Versus Not Treated With Medication

	OR (95% CI)	*p* Value
Age at diagnosis (+1 year)	1.01 (1.00–1.02)	**<0.0001**
Sex (ref: male)		
Female	1.99 (1.98–2.00)	**<0.0001**
Race (ref: White)		
Black	0.85 (0.84–0.86)	**<0.0001**
Hispanic	1.37 (1.36–1.38)	**<0.0001**
Asian	1.65 (1.63–1.67)	**<0.0001**
Other/unknown	0.52 (0.39–0.69)	**<0.0001**
Region (ref: South)		
Northeast	0.74 (0.73–0.75)	**<0.0001**
Midwest	0.87 (0.86–0.88)	**<0.0001**
West	1.34 (1.33–1.35)	**<0.0001**
Unknown	0.67 (0.66–0.68)	**<0.0001**
Education level (ref: less than 12th grade)		
High school diploma	0.86 (0.85–0.87)	**<0.0001**
Less than bachelor's	0.86 (0.85–0.87)	**<0.0001**
Bachelor's degree plus	0.84 (0.83–0.85)	**<0.0001**
Unknown	0.87 (0.86–0.88)	**<0.0001**
Net worth (ref: <25 K)		
25 K–149 K	0.93 (0.91–0.95)	**<0.0001**
150 K–249 K	0.93 (0.91–0.95)	**<0.0001**
250 K–499 K	0.97 (0.95–0.99)	**<0.0001**
>500 K	1.05 (1.04–1.06)	**<0.0001**
Unknown	0.79 (0.77–0.81)	**<0.0001**
CCI (+1)	1.03 (1.02–1.04)	**<0.0001**

*Note*: Bold values indicate *p*‐values < 0.05.

Abbreviations: CCI = Charlson Comorbidity Index; CI = confidence interval; OR = odds ratio.

Among patients who received medication, 31,112 patients (5.0%) received anabolic medication. Patients who were younger, male, White, from the Southern US, had higher education level or net worth, or had greater comorbidity burden were more likely to receive anabolic medication prescriptions (Supplemental Table S3). Upon multivariable logistic regression, decreased age, White race, completion of at least a bachelor's degree, higher net worth, and greater comorbidity burden were independent predictors of receiving anabolic medication (Table 2).

**Table 2:** Multivariable Logistic Regression of Patients Receiving Anabolic Versus Antiresorptive Osteoporosis Medications

	OR (95% CI)	*p* Value
Age at diagnosis (+1 year)	0.98 (0.97–0.99)	**<0.0001**
Sex (ref: male)		
Female	0.90 (0.89–1.11)	0.1617
Race (ref: White)		
Black	0.67 (0.62–0.74)	**<0.0001**
Hispanic	0.75 (0.70–0.80)	**0.0148**
Asian	0.83 (0.75–0.91)	0.4821
Other/unknown	0.84 (0.78–1.08)	0.3332
Region (ref: South)		
Northeast	0.44 (0.38–0.51)	0.8362
Midwest	0.91 (0.84–0.98)	0.8801
West	0.60 (0.55–0.65)	0.8547
Unknown	0.78 (0.49–1.24)	0.8707
Education level (ref: less than 12th grade)		
High school diploma	1.32 (1.22–1.43)	0.4932
Less than bachelor's	1.38 (1.28–1.50)	0.1389
Bachelor's degree plus	1.65 (1.52–1.79)	**<0.0001**
Unknown	1.09 (0.62–1.91)	0.0675
Net worth (ref: <25 K)		
25 K–149 K	1.06 (0.99–1.13)	0.3946
150 K–249 K	1.01 (1.00–1.02)	**0.0165**
250 K–499 K	1.08 (0.93–1.24)	0.8756
>500 K	1.19 (1.10–1.28)	**0.0044**
Unknown	1.15 (0.98–1.37)	0.0578
CCI (+1)	1.02 (1.01–1.03)	**<0.0001**

*Note*: Bold values indicate *p*‐values < 0.05.

Abbreviations: CCI = Charlson Comorbidity Index; CI = confidence interval; OR = odds ratio.

**Fig. 1:**
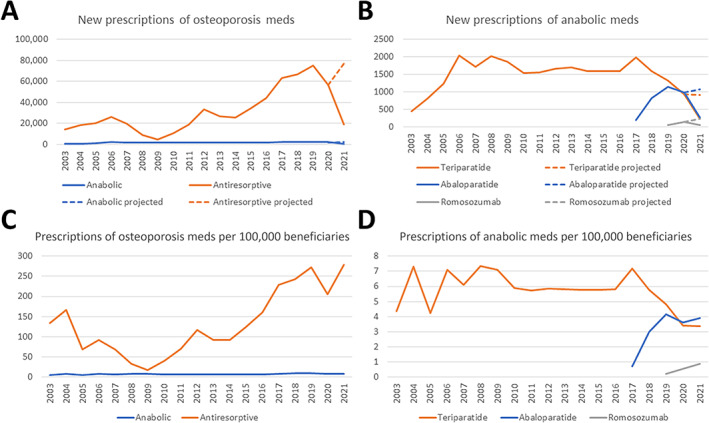
Trends in osteoporosis medication prescription. New prescriptions of (*A*) overall osteoporosis medications and (*B*) anabolic medications, and prescriptions per 100,000 beneficiaries of (*C*) overall osteoporosis medications and (*D*) anabolic medications, 2003–2021, stratified by medication type. Prescription data from January to March 2021 was used to create projections for a full year.

The total number of osteoporosis medication prescriptions increased more than fourfold from 14,484 in 2003 to 58,383 in 2020, with a projected number of 79,372 in 2021 (448% increase). The number of anabolic medication prescriptions increased from 458 in 2003 to 2053 in 2020, with a projected number of 2240 in 2021 (389% increase). Of the anabolic medications, teriparatide prescriptions increased from 458 in 2003 to 926 in 2020, with a projected number of 924 in 2021. Of note, teriparatide prescriptions peaked in 2017 at 1196. Abaloparatide prescriptions increased from 199 in 2017 to 984 in 2020, with a projected number of 1076 in 2021 (440% increase). Romosozumab prescriptions increased from 58 in 2019 to 143 in 2020 with a projected number of 240 in 2021 (314% increase) (Fig. 1).

The proportion of anabolic medication prescriptions remained roughly stable, from 3.2% in 2003 to 2.8% in 2021, with a peak in 2009 at 28.7% of all osteoporosis medication prescriptions. Among the anabolic medications, the proportion of abaloparatide prescriptions increased from 9.1% in 2017 to 48.0% in 2021, whereas the proportion of romosozumab prescriptions increased from 2.3% in 2019 to 10.7% in 2021 (Supplemental Fig. S1).

**Fig. 2:**
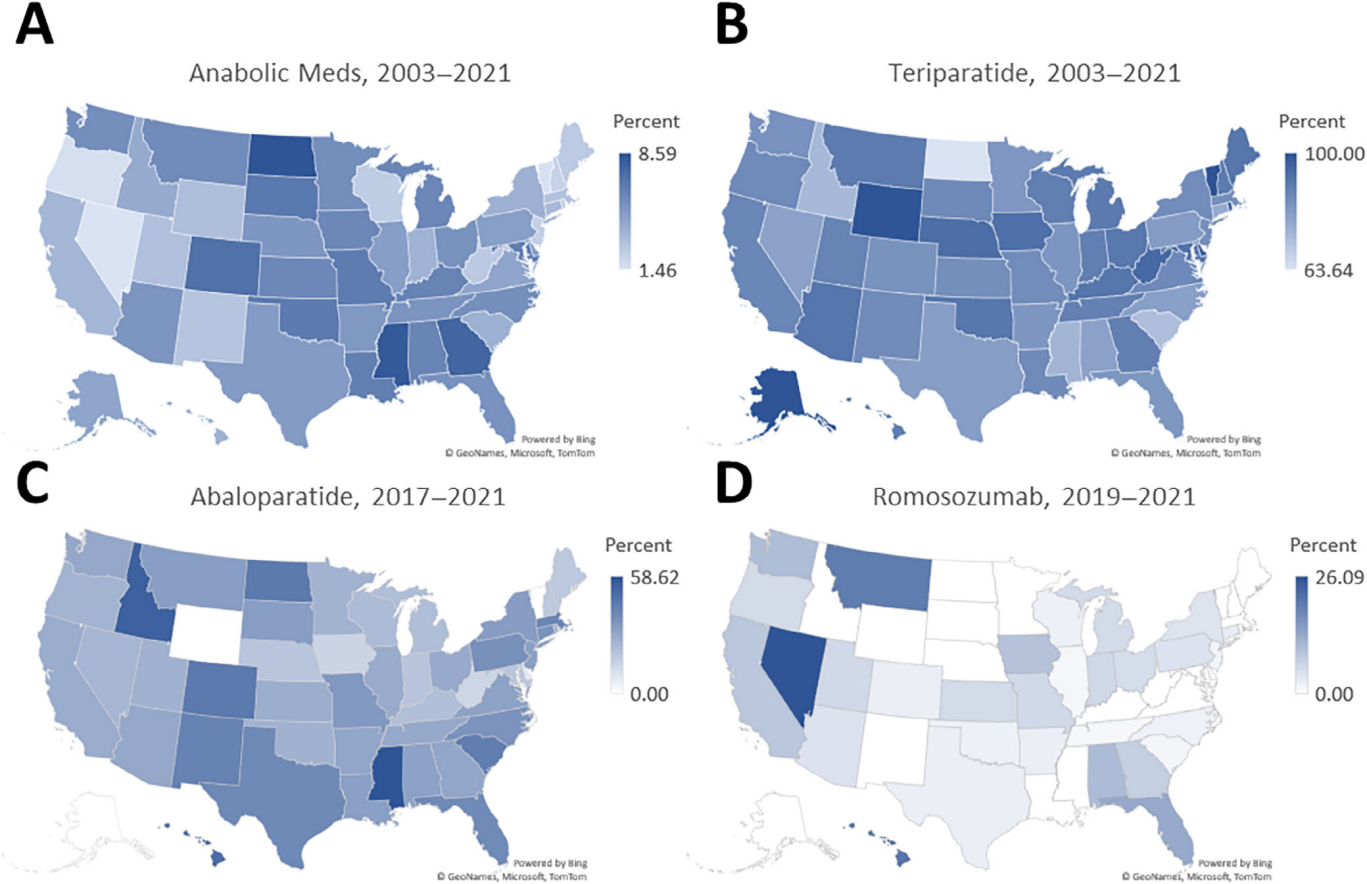
Geographic distribution of anabolic osteoporosis medication prescription. (*A*) Proportion of osteoporosis medications consisting of anabolic prescriptions. Proportion of anabolic prescriptions consisting of (*B*) teriparatide, (*C*) abaloparatide, and (*D*) romosozumab prescriptions.

There was significant geographic variation in prescription of anabolic medications. Among those prescribed osteoporosis medications, the states with the highest proportion of anabolic medication prescriptions were North Dakota (8.59%), Mississippi (8.36%), and Georgia (7.71%); lowest were Nevada (1.46%), Vermont (1.54%), and Oregon (1.67%). Among those prescribed anabolic medications, the states with the highest proportion of teriparatide prescriptions were Alaska, Vermont, and Wyoming (all 100%); lowest were North Dakota (63.6%), South Carolina (73.0%), and Idaho (74.4%). From 2017 to 2021, the states with the highest proportion of abaloparatide prescriptions were Mississippi (58.6%), Idaho (54.4%), and Hawaii (50.0%); lowest were Alaska, Vermont, and Wyoming (all 0%). From 2019 to 2021, the states with the highest proportion of romosozumab prescriptions were Nevada (26.1%), Hawaii (21.1%), and Montana (20.0%); there were several states with no prescriptions (Fig. 2).

In addition, there was variation in the types of providers prescribing anabolic medications. Anabolic medications were most commonly prescribed by rheumatologists (18.5%), endocrinologists (16.8%), and general internists (15.3%; Table 3).

**Table 3:** Types of Providers Prescribing Anabolic Osteoporosis Medications

Type of prescriber	Frequency (%)
Rheumatologist	5761 (18.5)
Endocrinologist	5228 (16.8)
General internist	4745 (15.3)
Family practitioner	3360 (10.8)
Orthopedic surgeon	1884 (6.1)
OB/GYN	677 (2.2)
Nurse practitioner	613 (2.0)
Physician's assistant	554 (1.8)
Nephrologist	365 (1.2)
Physical therapist	311 (1.0)
Neurosurgeon	294 (0.9)
IM specialist	154 (0.5)
Geriatric medicine	92 (0.3)

Abbreviation: IM = internal medicine.

Over the study period, the median inflation‐adjusted OOP monthly cost for anabolic medications ($37.60, interquartile range [IQR]: $79.23) was $17 higher than that for antiresorptive medications ($20.27, IQR: $33.71, *p* < 0.0001). OOP monthly cost varied significantly among the anabolic medications, as median OOP cost of abaloparatide was $10 higher than that of romosozumab and $23 higher than that of teriparatide. OOP monthly cost also varied significantly among the antiresorptive medications; notably, denosumab carried the highest median OOP cost at over $100 monthly (IQR: $115.79), and among the bisphosphonates, zoledronate carried the highest median OOP cost at nearly $59 monthly (IQR: $101.29) (Table 4).

**Table 4:** Median Inflation‐Adjusted Out‐of‐Pocket Monthly Costs of Anabolic and Antiresorptive Osteoporosis Medications (in US Dollars)

	Median (IQR)	*p* Value
Anabolic	$37.60 (79.23)	**<0.0001**
Antiresorptive	$20.27 (33.71)	
Teriparatide	$37.20 (71.88)	**<0.0001**
Abaloparatide	$60.85 (105.92)	
Romosozumab	$50.68 (97.58)	
Alendronate	$15.44 (32.13)	**<0.0001**
Calcitonin	$32.97 (42.31)	
Denosumab	$103.98 (115.79)	
Etidronate	$11.97 (10.70)	
Ibandronate	$16.56 (31.09)	
Pamidronate	$3.87 (35.34)	
Raloxifene	$29.39 (40.23)	
Risedronate	$45.12 (43.89)	
Zoledronate	$58.82 (101.29)	

*Note*: Bold values indicate *p*‐values < 0.05.

Abbreviation: IQR = interquartile range.

**Fig. 3:**
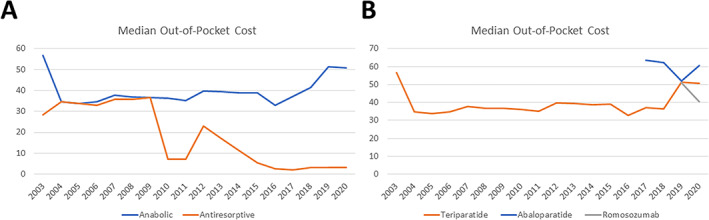
Out‐of‐pocket costs of osteoporosis medication prescription. Median inflation‐adjusted out‐of‐pocket costs in US dollars for (*A*) anabolic versus antiresorptive osteoporosis medications and (*B*) stratified anabolic medications, 2003–2020.

From 2003 to 2020, median OOP cost for antiresorptive medications decreased significantly from $28.52 to $3.07 (CAGR: −12.3%), whereas median OOP cost for anabolic medications only decreased from $56.69 to $50.78 (CAGR: −0.6%), with a nadir of $32.91 in 2016. Concordantly, from 2003 to 2020, median OOP cost for teriparatide decreased from $56.69 to $50.62 (CAGR: −0.6%). After its approval in 2017, median OOP for abaloparatide decreased from $63.49 to $60.43 (CAGR: −1.2%), with a nadir of $51.93 in 2019. After its approval in 2019, median OOP cost for romosozumab decreased from $51.02 to $40.31 (Fig. 3). Among the antiresorptive medications, trends in median OOP costs varied widely but overall decreased over the study period (Supplemental Fig. S2, Supplemental Table S4).

Median length of time spent taking anabolic medications was less than a year, at 103.5 (IQR: 238.5 days), 137 (IQR: 379 days), and 233 days (IQR 589 days) for teriparatide, abaloparatide, and romosozumab, respectively. The distributions of length of time spent taking osteoporosis medications were all right‐skewed (Table 5, Supplemental Fig. S3, Supplemental Table S5).

**Table 5:** Median Number of Days Taking Osteoporosis Medication

	Median (IQR)
Alendronate	274 (697)
Calcitonin	69 (398)
Denosumab	217 (720)
Etidronate	225 (634)
Ibandronate	181 (567)
Pamidronate	76 (367)
Raloxifene	398 (1059)
Risedronate	237 (634)
Zoledronate	30 (375)
Teriparatide	103.5 (238.5)
Abaloparatide	137 (379)
Romosozumab	233 (589)

Abbreviation: IQR = interquartile range.

**Fig. 4:**
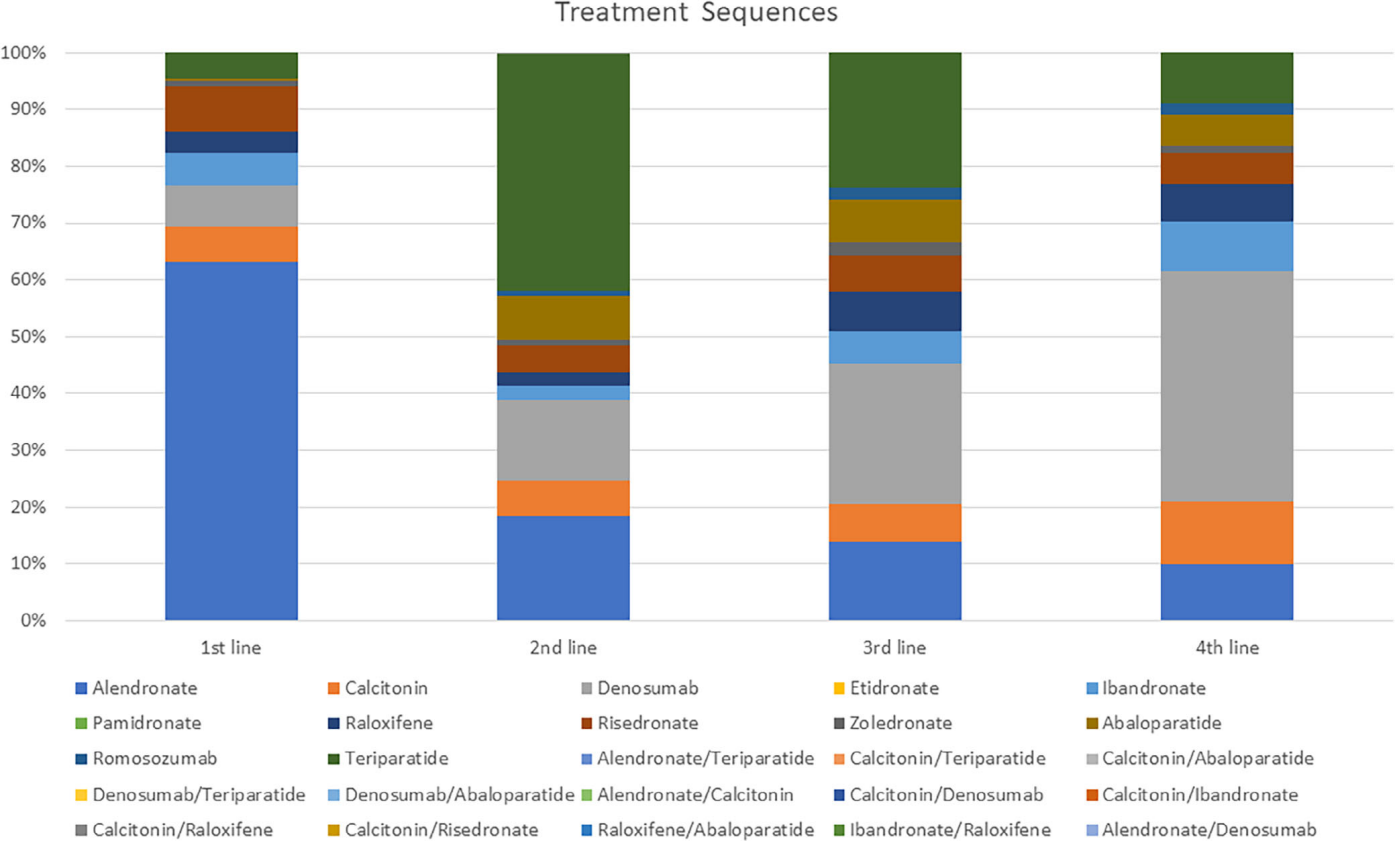
Osteoporosis treatment pathways. Stacked‐bar diagram demonstrating distribution of first to forth‐line osteoporosis medications prescribed.

The sequence of osteoporosis medications trialed by each patient varied widely. A total of 389,772 patients (63.2%) were prescribed alendronate and no other medications. Only 8388 patients (1.4%) tried two or more medications over the study period. Thirty‐four patients were prescribed two osteoporosis medications simultaneously (Fig. 4, Supplemental Table S6).

## Discussion

In this study, we demonstrate that overall osteoporosis medication prescription has increased more than fourfold from 2003 to 2021. Although anabolic medication prescription also increased significantly, it did not match this pace (Fig. 1*A*). Additionally, only a minority (5%) of patients are prescribed anabolic osteoporosis medications. There was a large decline of new prescriptions of antiresorptive medications beginning in 2006, with a nadir in 2009, which coincides with the first reports of atypical femur fractures with long‐term bisphosphonate use.^(^ ^17^, ^18^ ^)^ However, after multiple additional studies published on this topic, the number of new prescriptions recovered by 2012.^(^ ^19^, ^20^ ^)^ The decrease in median out‐of‐pocket costs for antiresorptive medications from 2009 through 2021 may explain the increase in prescriptions (Fig. 3). New anabolic medication prescriptions were relatively steady over this period, with a spike observed beginning in 2017, with FDA approval of abaloparatide. Concordantly, new abaloparatide prescriptions outpaced teriparatide and are projected to be greater in number in 2021 (Fig. 1*B*). Decreases in new prescriptions in 2020 may be due to the effects of the COVID‐19 pandemic interrupting access to care and delaying care for non‐COVID‐19‐related and non‐emergent medical care.^(^ ^21^ ^)^

Overall, female patients were nearly twice as likely to receive osteoporosis treatment compared with their male counterparts. Older patients were also more likely to receive osteoporosis medication prescriptions. For every 1‐year increase in a patient's age, the likelihood of receiving treatment increased 1%. In addition, patients of a higher net worth were more likely to receive treatment; however, higher education level did not have the same effect. Furthermore, Hispanic or Asian patients were more likely to receive treatment than White patients (Table 1), similar to trends observed in the Medicare Part D patient population,^(^ ^22^ ^)^ as well a recent study on fracture prevention for nursing home patients with dementia.^(^ ^23^ ^)^

Anabolic medications were more often prescribed to younger patients. Patients on anabolic medications were also more likely to be White, have higher educational status, and have higher net worth (Table 2). One potential explanation for this socioeconomic difference is that anabolic medications are generally newer and overall a more expensive class of drugs. More specifically, median inflation‐adjusted OOP monthly costs for anabolic medications were $17 higher than that for antiresorptive medications. However, this cost difference is quite small and is unlikely to fully explain the difference in prescription patterns. Unfortunately, our finding is consistent with other work showing that higher standards of care are disproportionately offered to patients from higher socioeconomic and educational backgrounds, leading to disparities in patient outcomes.^(^ ^24^, ^25^, ^26^ ^)^ For example, referral to and surgery at high‐volume hospitals may offer higher standards of care and fewer postoperative complications, yet these have been shown to be disproportionately offered to White and higher socioeconomic status patients.^(^ ^27^ ^)^ Additional studies are necessary to identify the factors underlying anabolic medication prescription trends and to increase the uptake of anabolic agents.

Another interesting finding is that the geographical spread of prescriptions of anabolic medications is more varied the longer the medication has been on the market. This is not surprising given the small number of overall prescriptions in this medication class. When the medication is brand‐new to the market, it is prescribed in only a few centers; as more data are published on its efficacy, its prescription increases across a larger geographic region. At the present time, the geographical distribution of romosozumab prescriptions is mostly limited to Western states (Nevada and Montana), as well as Iowa and a select few Southeastern states (Florida, Georgia, and Alabama), which is also consistent with a recent study on fracture prevention for nursing home patients with dementia.^(^ ^23^ ^)^

Not surprisingly, the top four types of providers who prescribe anabolic medications are rheumatologists, endocrinologists, general internists, and family practitioners, ie, osteoporosis specialists and primary care providers. This trend indicates that it is important for providers who often interact with this patient population, such as orthopedic and neurological surgeons, to collaborate with these specialists to optimize treatment and long‐term follow‐up of osteoporosis. Indeed, some institutions have established a “fracture liaison service” to enhance collaboration between orthopedic/neurosurgeons and bone specialists to decrease risk of future fragility fractures. These services also provide perioperative BMD optimization to reduce risk of postoperative complications such as pseudarthrosis and hardware failure.

Another important finding is that most patients do not switch medications once they have an established regimen (Fig. 4, Supplemental Table S6). In this database, only 1.4% of patients trial more than one medication. Published literature shows sustained recoveries in bone in patients who were treated with anabolic therapy followed by antiresorptive therapy but not the reverse.^(^ ^10^, ^11^ ^)^ In our cohort, only 3624 patients (0.6%) underwent this recommended treatment sequence. This suggests that many US osteoporotic patients may be getting suboptimal care, which could potentially be improved with increased multidisciplinary collaboration or referral to appropriate specialists to better manage and follow up osteoporosis treatment in the long term.

This study has multiple limitations. It is a descriptive, retrospective study, so we are limited in causal inference. Given only certain variables are available in the data set, it is not possible to definitively identify the causes underlying all of our observed trends. As a result, we present possible explanations for the observed trends that are sometimes speculative but are supported by prior studies. The data set does not provide certain information such as dual‐energy X‐ray absorptiometry (DXA) scan results (bone density *T*‐scores or *Z*‐scores), which are important to evaluate the degree and severity of osteoporosis. In addition, there is also missing/unknown data for certain variables, although this constitutes only a small proportion of our cohort (Supplemental Tables S2 and S3). This study also did not analyze the rates of fragility fracture among patients treated with anabolic versus antiresorptive medications, or outcomes after patients with recent fragility fracture are started on anabolic versus antiresorptive agents. These are important questions that should be the topic of future study. Finally, our data are obtained from CDM, which constitutes privately insured and Medicare Advantage beneficiaries from a single payer and may not be generalized to all patient populations. This is particularly relevant with regard to OOP payments for patients, which can vary greatly depending on insurance status. Despite these limitations, we believe our study offers valuable insight on national trends in osteoporosis treatment and specifically variation in anabolic medication prescription in a very large patient population.

In summary, although new prescriptions of osteoporosis medications increased more than fourfold from 2003 to 2021, anabolic medication prescriptions did not match this increase. Anabolic medications for osteoporosis management are more often prescribed to White and higher socioeconomic status patients. Median out‐of‐pocket costs are higher for these medications, which may partially explain these differences. Lastly, although data suggest that prescribing antiresorptive medications after a course of anabolic medications provides better outcomes, this treatment sequence occurred in only 0.6% of patients.

## Author Contributions

**Harsh Wadhwa:** Data curation; formal analysis; visualization; writing – original draft; writing – review and editing. **Janet Yuling Wu:** Project administration; writing – original draft; writing – review and editing. **Jennifer S. Lee:** Conceptualization; supervision. **Corinna C. Zygourakis:** Conceptualization; supervision; writing – original draft; writing – review and editing.

## Disclosures

CCZ reports consulting for Stryker and 7D; speaking and teaching arrangements with Stryker, 7D, and Amgen; and support from Stryker for an unrelated research study to support a clinical research coordinator. None of these relationships or financial activities pertain to this article. All other authors do not report any disclosures or conflicts of interest.

### Peer Review

The peer review history for this article is available at https://www.webofscience.com/api/gateway/wos/peer-review/10.1002/jbm4.10800.

## Supporting information


**Fig. S1.** Trends in osteoporosis medication prescription. Proportion of new prescriptions of (*A*) overall osteoporosis medications and (*B*) anabolic medications, 2003–2021, stratified by medication type.
**Fig. S2.** Out‐of‐pocket costs in US dollars of antiresorptive osteoporosis medication prescriptions. Median inflation‐adjusted out‐of‐pocket costs for stratified antiresorptive osteoporosis medications, 2003–2020.
**Fig. S3.** Length of time taking osteoporosis medications. Histograms depicting distribution of number of days spent taking medications, calculated via quantity of medication prescribed.
**Table S1.** International Classification of Diseases, Ninth Revision (ICD‐9), International Classification of Diseases, Tenth Revision (ICD‐10), and National Drug Code (NDC) Codes Used to Identify Patients
**Table S2.** Demographic and Socioeconomic Characteristics of Patients With Osteoporosis Treated Versus Not Treated With Medication
**Table S3.** Demographic and Socioeconomic Characteristics of Patients Receiving Anabolic Versus Antiresorptive Osteoporosis Medications
**Table S4.** Out‐of‐Pocket Costs in US Dollars of Antiresorptive Osteoporosis Medication Prescriptions; Median Inflation‐Adjusted Out‐of‐Pocket Costs for Stratified Antiresorptive Osteoporosis Medications, 2003–2020
**Table S5.** Length of Time Taking Osteoporosis Medications; Quantitative Depiction of Distribution of Number of Days Spent Taking a Medication (Cells With <11 Patients Were Censored Due to Data Requirements)
**Table S6.** Osteoporosis Treatment Pathways Demonstrating Distribution of First to Fourth‐Line Osteoporosis Medications Prescribed (Cells With <11 Patients Were Censored Due to Data Requirements)
