# Supplementary material for: Anabolic and Antiresorptive Osteoporosis Treatment: Trends, Costs, and Sequence in a Commercially Insured Population, 2003–2021
Source: JBMR Plus. 2023 Jul 24;7(10):e10800. doi: 10.1002/jbm4.10800 (PMC10556263; doi:10.1002/jbm4.10800)
Supplement: Supplementary file 1 — Fig. S1. Trends in osteoporosis medication prescription. Proportion of new prescriptions of (A) overall osteoporosis medications and (B) anabolic medications, 2003–2021, stratified by medication type. Fig. S2. Out‐of‐pocket costs in US dollars of antiresorptive osteoporosis medication prescriptions. Median inflation‐adjusted out‐of‐pocket costs for stratified antiresorptive osteoporosis medications, 2003–2020. Fig. S3. Length of time taking osteoporosis medications. Histograms depicting distribution of number of days spent taking medications, calculated via quantity of medication prescribed. Table S1. International Classification of Diseases, Ninth Revision (ICD‐9), International Classification of Diseases, Tenth Revision (ICD‐10), and National Drug Code (NDC) Codes Used to Identify Patients Table S2. Demographic and Socioeconomic Characteristics of Patients With Osteoporosis Treated Versus Not Treated With Medication Table S3. Demographic and Socioeconomic Characteristics of Patients Receiving Anabolic Versus Antiresorptive Osteoporosis Medications Table S4. Out‐of‐Pocket Costs in US Dollars of Antiresorptive Osteoporosis Medication Prescriptions; Median Inflation‐Adjusted Out‐of‐Pocket Costs for Stratified Antiresorptive Osteoporosis Medications, 2003–2020 Table S5. Length of Time Taking Osteoporosis Medications; Quantitative Depiction of Distribution of Number of Days Spent Taking a Medication (Cells With <11 Patients Were Censored Due to Data Requirements) Table S6. Osteoporosis Treatment Pathways Demonstrating Distribution of First to Fourth‐Line Osteoporosis Medications Prescribed (Cells With <11 Patients Were Censored Due to Data Requirements) [file JBM4-7-e10800-s001.docx]

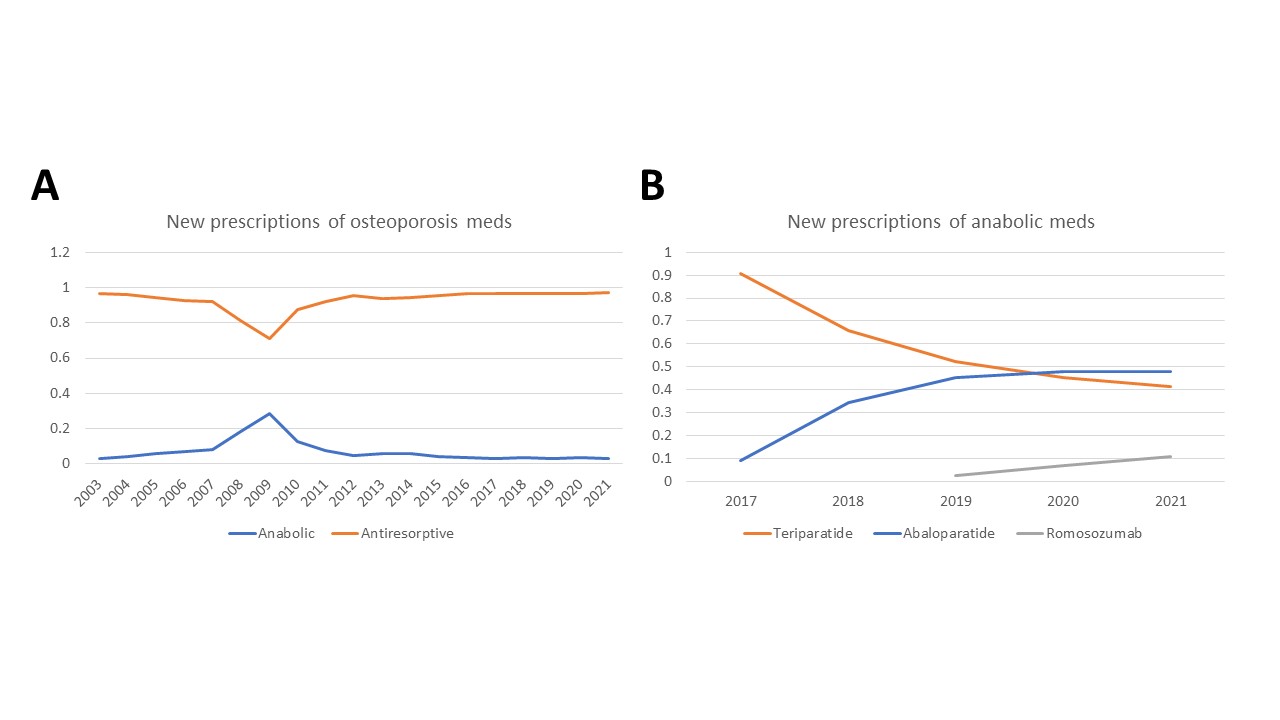


Supplemental Figure 1. Trends in osteoporosis medication prescription. Proportion of new prescriptions of (A) overall osteoporosis medications and (B) anabolic medications, 2003-2021, stratified by medication type.


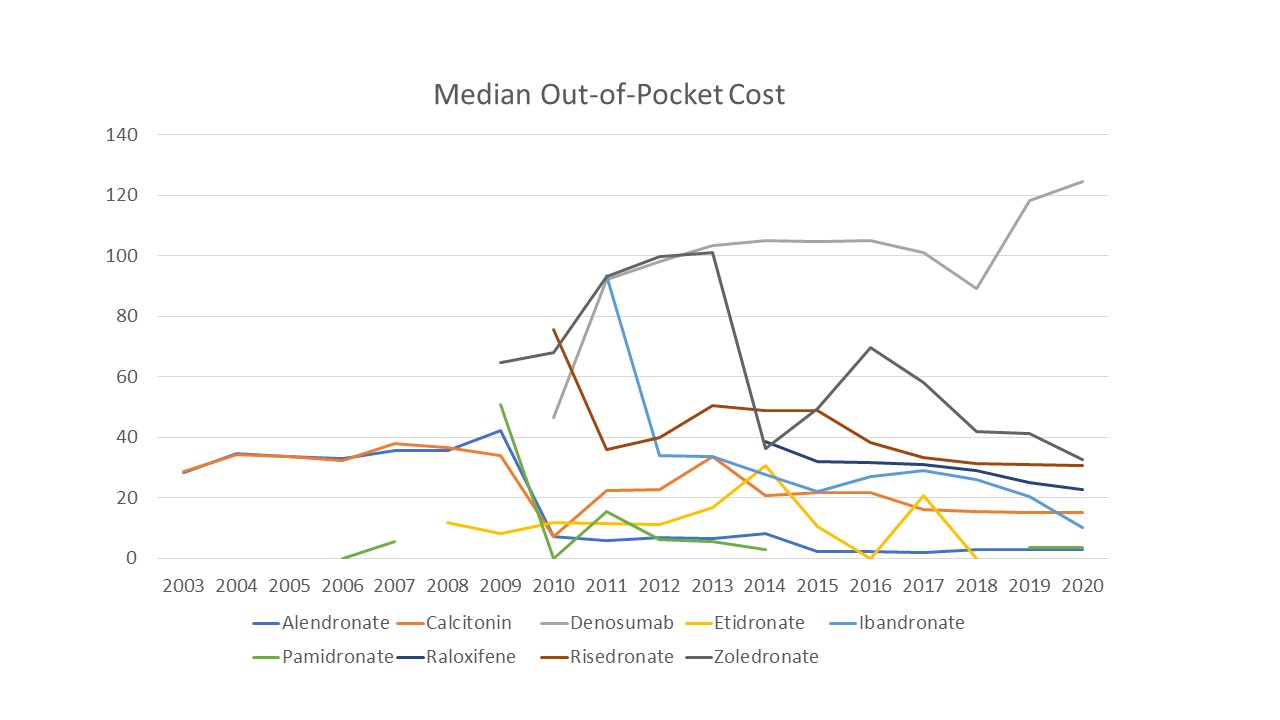


Supplemental Figure 2. Out-of-pocket costs in US dollars of antiresorptive osteoporosis medication prescriptions. Median inflation-adjusted out-of-pocket costs for stratified antiresorptive osteoporosis medications, 2003-2020


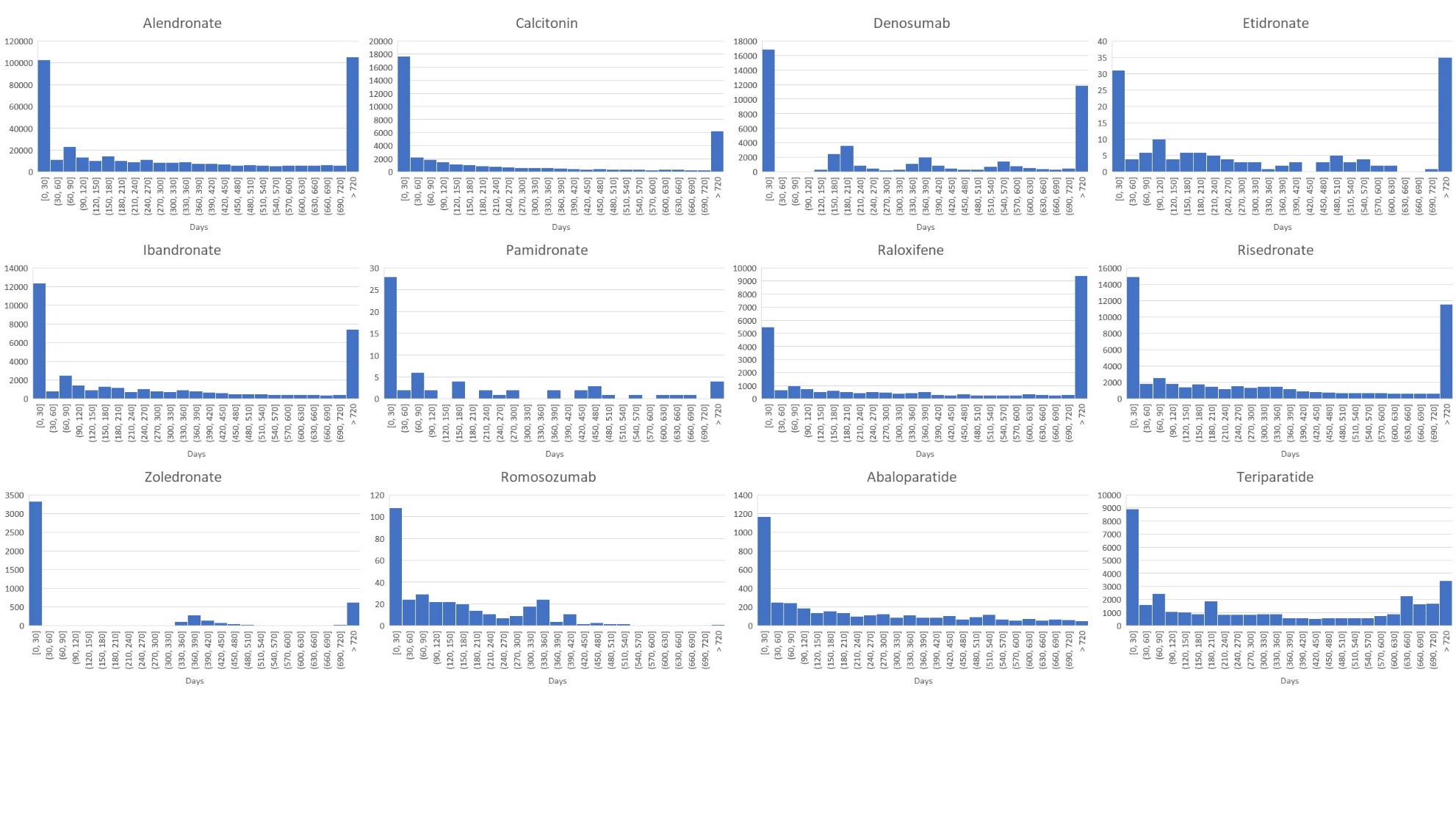


Supplemental Figure 3. Length of time taking osteoporosis medications. Histograms depicting distribution of number of days spent taking medications, calculated via quantity of medication prescribed.

Supplemental Table 1. International Classification of Diseases, Ninth Revision (ICD-9), International Classification of Diseases, Tenth Revision (ICD-10), and National Drug Code (NDC) codes used to identify patients

| Identifier | Relevant Codes |
| --- | --- |
| Osteoporosis | 733.0, 733.00, 733.01, 733.02, 733.03, 733.09, M80, M80.0, M80.00, M80.00XA, M80.00XD, M80.00XG, M80.00XK, M80.00XP, M80.00XS, M80.01, M80.011, M80.011A, M80.011D, M80.011G, M80.011K, M80.011P, M80.011S, M80.012, M80.012A, M80.012D, M80.012G, M80.012K, M80.012P, M80.012S, M80.019, M80.019A, M80.019D, M80.019G, M80.019K, M80.019P, M80.019S, M80.02, M80.021, M80.021A, M80.021D, M80.021G, M80.021K, M80.021P, M80.021S, M80.022, M80.022A, M80.022D, M80.022G, M80.022K, M80.022P, M80.022S, M80.029, M80.029A, M80.029D, M80.029G, M80.029K, M80.029P, M80.029S, M80.03, M80.031A, M80.031D, M80.031G, M80.031K, M80.031P, M80.031S, M80.032, M80.032A, M80.032D, M80.032G, M80.032K, M80.032P, M80.032S, M80.039, M80.039A, M80.039D, M80.039G, M80.039K, M80.039P, M80.039S, M80.04, M80.041, M80.041A, M80.041D, M80.041G, M80.041K, M80.041P, M80.041S, M80.042, M80.042A, M80.042D, M80.042G, M80.042K, M80.042P, M80.042S, M80.049, M80.049A, M80.049D, M80.049G, M80.049K, M80.049P, M80.049S, M80.05, M80.051, M80.051A, M80.051D, M80.051G, M80.051K, M80.051P, M80.051S, M80.052, M80.052A, M80.052D, M80.052G, M80.052K, M80.052P, M80.052S, M80.059, M80.059A, M80.059D, M80.059G, M80.059K, M80.059P, M80.059S, M80.06, M80.061, M80.061A, M80.061D, M80.061G, M80.061K, M80.061P, M80.061S, M80.062, M80.062A, M80.062D, M80.062G, M80.062K, M80.062P, M80.062S, M80.069, M80.069A, M80.069D, M80.069G, M80.069K, M80.069P, M80.069S, M80.07, M80.071, M80.071A, M80.071D, M80.071G, M80.071K, M80.071P, M80.071S, M80.072, M80.072A, M80.072D, M80.072G, M80.072K, M80.072P, M80.072S, M80.079, M80.079A, M80.079D, M80.079G, M80.079K, M80.079P, M80.079S, M80.08, M80.0XA, M80.0XD, M80.0XG, M80.0XK, M80.0XP, M80.0XS, M80.0A, M80.0AXA, M80.0AXD, M80.0AXG, M80.0AXK, M80.0AXP, M80.0AXS, M80.8, M80.80, M80.80XA, M80.80XD, M80.80XG, M80.80XK, M80.80XP, M80.80XS, M80.81, M80.811, M80.811A, M80.811D, M80.811G, M80.811K, M80.811P, M80.811S, M80.812, M80.812A, M80.812D, M80.812G, M80.812K, M80.812P, M80.812S, M80.819, M80.819A, M80.819D, M80.819G, M80.819K, M80.819P, M80.819S, M80.82, M80.821, M80.821A, M80.821D, M80.821G, N80821K, M80.821P, M80.821S, M80.822, M80.822A, M80.822D, M80.822G, M80.822K, M80.822P, M80.822S, M80.829, M80.829A, M80.829D, M80.829G, M80.829K, M80.829P, M80.829S, M80.83, M80.831, M80.831A, M80.831D, M80.831G, M80.831K, M80.831P, M80.831S, M80.832, M80.832A, M80.832D, M80.832G, M80.832K, M80.832P, M80.832S, M80.839, M80.839A, M80.839D, M80.839G, M80.839K, M80.839P, M80.839S, M80.84, M80.841, M80.841A, M80.841D, M80.841G, M80.841K, M80.841P, M80.841S, M80.842, M80.842A, M80.842D, M80.842G, M80.842K, M80.842P, M80.842S, M80.849, M80.849A, M80.849D, M80.849G, M80.849K, M80.849P, M80.849S, M80.85, M80.851, M80.851A, M80.851D, M80.851G, M80.851K, M80.851P, M80.851S, M80.852, M80.852A, M80.852D, M80.852G, M80.852K, M80.852P, M80.852S, M80.859, M80.859A, M80.859D, M80.859G, M80.859K, M80.859P, M80.859S, M80.86, M80.861, M80.861A, M80.861D, M80.861G, M80.861K, M80.861P, M80.861S, M80.862, M80.862A, M80.862D, M80.862G, M80.862K, M80.862P, M80.862S, M80.869, M80.869A, M80.869D, M80.869G, M80.869K, M80.869P, M80.869S, M80.87, M80.871, M80.871A, M80.871D, M80.871G, M80.871K, M80.871P, M80.871S, M80.872, M80.872A, M80.872D, M80.872G, M80.872K, M80.872P, M80.872S, M80.879, M80.879A, M80.879D, M80.879G, M80.879K, M80.879P, M80.879S, M80.88, M80.88XA, M80.88XD, M80.88XG, M80.88XK, M80.08XP, M80.08XS, M80.8A, M80.8AXA, M80.8AXD, M80.8AXG, M80.8AXK, M80.8AXP, M80.8AXS, M81, M81.0, M81.6, M81.8, Z87.310 |
| Alendronate | 69543013004, 69543013012, 69543013020, 69543013104, 69543013112, 69543013120, 60723020531, 60723020532, 60723020533, 60723020731, 60723020732, 10135069612, 10135069744, 10135071812, 10135071944, 73309003401, 73309003402, 69543013004, 69543013112, 54028259, 51224030110, 115167601, 115167926, 65862032701, 65862032804, 69097022316, 60429005572, 16714063101, 16714063201, 64980034001, 64980034214, 50090035930, 63629073291, 68071040174, 33261099301, 53217032101, 33261082001, 53217032101, 50090023220, 50090010910, 50090040760, 50090018020, 50090046920, 50090031260, 55695001200, 61919068904, 61919072904, 68258030141, 66116042804, 68071031714, 68071015144, 68071033404, 68071041201, 54868586000, 54868586200, 68788071304, 71205004804, 21695090104, 70518000330, 54868385700, 54868446300, 17139040001, 178010101, 6003144, 6027001, 54868548000, 60723020733, 10135069644, 10135071844, 69543013012, 69543013120, 115167608, 115167930, 65862032710, 65862032808, 69097022376, 60429005574, 16714063102, 16714063202, 64980034003, 10135069712, 10135071912, 69543013020, 115167801, 115167934, 65862032730, 65862032810, 69097022416, 60429005672, 16714063110, 16714063210, 64980034112, 50090010951, 50090018030, 54868586100, 54868446200, 6027044, 10135069720, 10135071920, 69543013104, 115167808, 115168008, 65862032799, 65862032820, 69097022476, 60429005674, 16714063111, 16714063211, 64980034114, 54868586110, 54868446210, 6071044, 115168126, 115168130, 115168134, 65862032904, 65862032908, 65862032910, 65862032920, 16714063301, 16714063302, 16714063310, 16714063311, 69543013004, 51138019020, 21695090104, 41616063768, 16714063201, 47335063768, 61919072904, 115167930, 69097022376, 76439013004, 555071954, 93517229, 378356899 |
| Calcitonin | 78014923, 78031154, 245000835, 781632079, 35207000305, 41701000501, 41701000503, 41701001002, 49884016111, 52416011101, 52416011601, 54766014923, 54868549900, 54868632300, 60505082300, 60505082306, 63586000201, 63629215201, 67457067502 |
| Denosumab | 55513071001, 55513073001 |
| Etidronate | 378328691, 378328891, 53296002800, 68151152109, 75839055199 |
| Ibandronate | 60505060970, 55150019183, 67457052433, 25021082761, 62756021840, 47781010307, 55111057503, 60505027950, 65862023703, 33342015001, 60429064373, 4019109, 4018683, 54868053220, 47781010321, 55111057511, 33342015052, 54868053221, 47781010333, 55111057513, 33342015053, 55111057543 |
| Pamidronate | 67457043010, 67457044610, 23360002310, 23360002410, 10139030610, 10139030710, 69010701, 69010901, 69018601, 517074501, 517074601, 703407551, 703408551, 25021080210, 25021080310, 53104759401, 55390012701, 55390012901, 55390020401, 59923060110, 59923060210, 59923060310, 61703032418, 61703032518, 61703032618, 61703035618, 62675071801, 63323073410, 63323073510, 67457043010, 67457044610, 67457047210, 67457061010 |
| Raloxifene | 69097082502, 31722025601, 71209008201, 65162005703, 65162005733, 65862070901, 65862070930, 43598050501, 76282025601, 76282025690, 66993066102, 50228030620, 93729001, 54868041700, 60687026611, 71335016631, 63629077081, 63629077085, 71610005360, 43353025360, 50090033630, 50090038660, 42291072610, 50268069411, 63629082091, 71335014601, 71335017151, 440529501, 440529581, 440529581, 2418402, 69097082507, 31722025610, 71209008205, 65162005709, 65162005750, 65862070903, 43598050510, 76282025605, 66993066130, 50228030630, 93729056, 54868041701, 60687026621, 71335016632, 63629077082, 50090033631, 50090038661, 42291072690, 50268069415, 63629082092, 71335014602, 71335017152, 440529505, 440529590, 440529590, 2418407, 69097082515, 31722025630, 71209008213, 65162005710, 65862070910, 43598050530, 76282025610, 66993066188, 63629077083, 440529530, 2418430, 65162005711, 65862070922, 76282025630, 63629077084, 440529560 |
| Risedronate | 115142634, 70771014364, 59762004074, 16714087001, 63304044009, 68382007573, 591204403, 591207539, 60505030962, 60505031652, 65862087003, 65862051710, 65862051810, 59762004054, 60723004101, 33342010706, 33342010807, 47335066608, 47335066708, 93309819, 93310056, 93777113, 430047015, 430047702, 430097903, 54868043860, 16714087002, 63304044011, 591204454, 591210230, 60505030972, 65862087011, 65862051722, 65862051830, 59762004055, 60723004104, 33342010707, 33342010815, 47335066618, 47335066718, 93309829, 93777119, 430047115, 430047801, 54868043861, 591206702, 591210930, 60505030974, 65862051730, 65862051878, 59762004061, 60723004112, 33342010749, 33342010937, 47335066683, 47335066783, 93309844, 93777179, 430047203, 430047802, 54868046710, 591207504, 60505031650, 65862051778, 65862051899, 59762004063, 33342010806, 33342010950, 47335066688, 47335066788, 93309956, 430047207, 54868060690, 65862051904, 65862051908, 47335066862, 47335066868, 47335072798, 47335092860, 47335092867 |
| Zoledronate | 16729024231, 68001043725, 51991006498, 63323096198, 68083014201, 68083025601, 16714081501, 55150028399, 17478032705, 60505061100, 55150026605, 68001036687, 68001036622, 54288010001, 51991006598, 43598033011, 55111068507, 68083011601, 23155017031, 409421501, 409421505, 50742041605, 67457039054, 67457092005, 25021080166, 143964201, 43598025552, 63323096600, 68083013501, 409422901, 409422801, 67457061910, 67457079410, 25021082667, 25021083082, 25021082682, 17478032491, 70860021051, 70860080282, 43598033111, 55111068852, 78043561, 17478032745, 68001036625 |
| Teriparatide | 52416012601, 47781065289, 2840001, 2840099 |
| Abaloparatide | 70539000101, 70539000102, 70539000198, 70539000199 |
| Romosozumab | 55513088001, 55513088002 |

Supplemental Table 2. Demographic and socioeconomic characteristics of patients with osteoporosis treated vs not treated with medication.

|  | Untreated | Treated | p-value |
| --- | --- | --- | --- |
| N | 2,372,191 | 616,635 |  |
| Age at diagnosis | 69 (18) | 69 (13) | **< 0.0001** |
| Sex |  |  | **< 0.0001** |
| Male | 326,858 (13.8) | 46,974 (7.6) |  |
| Female | 2,045,333 (86.2) | 569,661 (92.4) |  |
| Race |  |  | **< 0.0001** |
| White | 1,630,575 (68.7) | 402,264 (65.2) |  |
| Black | 212,836 (9.0) | 46,341 (7.5) |  |
| Hispanic | 232,121 (9.8) | 83,103 (13.5) |  |
| Asian | 85,068 (3.6) | 37,911 (6.2) |  |
| Other/Unknown | 211,591 (8.9) | 47,016 (7.6) |  |
| Region |  |  | **< 0.0001** |
| Northeast | 362,999 (15.3) | 69,706 (11.3) |  |
| Midwest | 471,041 (19.9) | 107,334 (17.4) |  |
| South | 1,023,011 (43.1) | 267,468 (43.4) |  |
| West | 508,663 (21.4) | 170,802 (27.7) |  |
| Unknown | 6,478 (0.3) | 1,325 (0.2) |  |
| Education Level |  |  | **< 0.0001** |
| Less than 12th Grade | 12,855 (0.5) | 4,723 (0.8) |  |
| High School Diploma | 600,102 (25.3) | 158,515 (25.7) |  |
| Less than Bachelor’s | 1,203,431 (50.7) | 315,895 (51.2) |  |
| Bachelor’s Degree Plus | 395,612 (16.7) | 102,087 (16.6) |  |
| Unknown | 160,191 (6.8) | 35,415 (5.7) |  |
| Net Worth |  |  | **< 0.0001** |
| <25K | 378,497 (16.0) | 107,617 (17.5) |  |
| 25K – 149K | 365,117 (15.4) | 94,302 (15.3) |  |
| 150K – 249K | 233,500 (9.8) | 58,139 (9.3) |  |
| 250K – 499K | 403,267 (17.0) | 102,707 (16.7) |  |
| >500K | 657,767 (27.7) | 183,083 (29.7) |  |
| Unknown | 334,043 (14.1) | 70,787 (11.5) |  |
| CCI | 3.64 (4.22) | 3.96 (4.42) | **< 0.0001** |

CCI = Charlson Comorbidity Index

Supplemental Table 3. Demographic and socio-economic characteristics of patients receiving anabolic vs. antiresorptive osteoporosis medications

|  | Antiresorptive | Anabolic | p-value |
| --- | --- | --- | --- |
| N | 585,523 | 31,112 |  |
| Age at diagnosis | 71 (13) | 67 (17) | **< 0.0001** |
| Sex |  |  | **< 0.0001** |
| Male | 44,012 (7.5) | 2,962 (9.5) |  |
| Female | 541,511 (92.5) | 28,150 (90.5) |  |
| Race |  |  | **< 0.0001** |
| White | 379,332 (64.8) | 22,932 (73.7) |  |
| Black | 44,496 (7.6) | 1,845 (5.9) |  |
| Hispanic | 80,269 (13.7) | 2,834 (9.1) |  |
| Asian | 36,722 (6.3) | 1,189 (3.8) |  |
| Other/Unknown | 44,704 (7.6) | 2,312 (7.4) |  |
| Region |  |  | **< 0.0001** |
| Northeast | 67,248 (11.5) | 2,458 (7.9) |  |
| Midwest | 101,762 (17.4) | 5,572 (17.9) |  |
| South | 251,803 (43.0) | 15,665 (50.3) |  |
| West | 163,404 (27.9) | 7,398 (23.8) |  |
| Unknown | 1,306 (0.2) | 19 (0.1) |  |
| Education Level |  |  | **< 0.0001** |
| Less than 12^th^ Grade | 4,596 (0.8) | 127 (0.4) |  |
| High School Diploma | 151,570 (25.9) | 6,945 (22.3) |  |
| Less than Bachelor’s | 300,166 (51.3) | 15,729 (50.6) |  |
| Bachelor’s Degree Plus | 95,444 (16.3) | 6,643 (21.4) |  |
| Unknown | 33,747 (5.8) | 1,668 (5.4) |  |
| Net Worth |  |  | **< 0.0001** |
| <25K | 103,046 (17.6) | 4,571 (14.7) |  |
| 25K – 149K | 89,832 (15.3) | 4,470 (14.4) |  |
| 150K – 249K | 55,272 (9.4) | 2,867 (9.2) |  |
| 250K – 499K | 97,352 (16.6) | 5,355 (17.2) |  |
| >500K | 172,858 (29.5) | 10,225 (32.9) |  |
| Unknown | 67,163 (11.5) | 3,624 (11.6) |  |
| CCI | 3.93 (4.41) | 4.26 (4.41) | **< 0.0001** |

CCI = Charlson Comorbidity Index

Supplemental Table 4. Out-of-pocket costs in US dollars of antiresorptive osteoporosis medication prescriptions. Median inflation-adjusted out-of-pocket costs for stratified antiresorptive osteoporosis medications, 2003-2020

|  | **2003** | **2004** | **2005** | **2006** | **2007** | **2008** | **2009** | **2010** | **2011** | **2012** | **2013** | **2014** |
| --- | --- | --- | --- | --- | --- | --- | --- | --- | --- | --- | --- | --- |
| **Alendronate** | $28.52 | $34.54 | $33.84 | $32.89 | $35.76 | $35.62 | $42.22 | $7.23 | $6.01 | $6.88 | $6.75 | $8.20 |
| **Calcitonin** | $28.69 | $34.36 | $33.86 | $32.47 | $37.88 | $36.77 | $34.06 | $7.24 | $22.63 | $22.88 | $33.77 | $20.71 |
| **Denosumab** |  |  |  |  |  |  |  | $46.69 | $92.34 | $98.24 | $103.33 | $104.90 |
| **Etidronate** |  |  |  |  |  | $12.06 | $8.28 | $11.94 | $11.54 | $11.34 | $16.92 | $30.82 |
| **Ibandronate** |  |  |  |  |  |  |  |  | $93.48 | $33.96 | $33.69 | $27.61 |
| **Pamidronate** |  |  |  |  | $5.65 |  | $50.86 | $0 | $15.61 | $6.30 | $5.66 | $2.83 |
| **Raloxifene** |  |  |  |  |  |  |  |  |  |  |  | $38.64 |
| **Risedronate** |  |  |  |  |  |  |  | $75.51 | $35.93 | $40.04 | $50.40 | $49.03 |
| **Zoledronate** |  |  |  |  | $40.23 |  | $64.83 | $68.07 | $93.31 | $99.79 | $101.16 | $36.39 |
| --------------------------------------------------------------------------------------------------------------------------------------------------------------- | | | | | | | | | | | | |
|  | **2015** | **2016** | **2017** | **2018** | **2019** | **2020** | **2021** |  |  |  |  |  |
| **Alendronate** | $2.21 | $2.19 | $2.15 | $3.12 | $3.07 | $3.03 | $2.94 |  |  |  |  |  |
| **Calcitonin** | $21.95 | $21.83 | $16.13 | $15.62 | $15.36 | $15.29 | $44.52 |  |  |  |  |  |
| **Denosumab** | $104.78 | $104.95 | $101.00 | $89.10 | $118.20 | $124.4 | $125.65 |  |  |  |  |  |
| **Etidronate** | $10.56 | $0 | $20.66 | $0 |  |  |  |  |  |  |  |  |
| **Ibandronate** | $22.07 | $27.17 | $29.20 | $26.26 | $20.62 | $10.16 | $10 |  |  |  |  |  |
| **Pamidronate** |  |  |  |  | $3.68 | $3.65 | $3.69 |  |  |  |  |  |
| **Raloxifene** | $32.09 | $31.72 | $30.95 | $29.20 | $25.01 | $22.91 | $30 |  |  |  |  |  |
| **Risedronate** | $48.89 | $38.35 | $33.38 | $31.53 | $30.94 | $30.73 | $44.19 |  |  |  |  |  |
| **Zoledronate** | $49.52 | $69.86 | $58.05 | $42.05 | $41.39 | $32.78 | $65.00 |  |  |  |  |  |

Supplemental Table 5. Length of time taking osteoporosis medications. Quantitative depiction of distribution of number of days spent taking a medication. Cells with <11 patients were censored due to data requirements.

|  | 0-60 | 60-120 | 120-180 | 180-240 | 240-300 | 300-360 | 360-420 | 420-480 | 480-540 | 540-600 | 600-660 | 660-720 | >720 |
| --- | --- | --- | --- | --- | --- | --- | --- | --- | --- | --- | --- | --- | --- |
| Alendronate | 114,137 | 36,145 | 25,104 | 19,426 | 20,071 | 18,034 | 14,998 | 13,319 | 12,900 | 11,677 | 11,657 | 12,297 | 105,655 |
| Calcitonin | 19,917 | 3,392 | 2,236 | 1,782 | 1,410 | 1,279 | 1,014 | 842 | 822 | 674 | 693 | 552 | 6,291 |
| Denosumab | 16,918 | 72 | 2,867 | 4,535 | 793 | 1,547 | 2,950 | 847 | 1,088 | 2,260 | 993 | 814 | 11,880 |
| Etidronate | 35 | 16 | <11 | 11 | <11 | <11 | <11 | <11 | <11 | <11 | <11 | <11 | 35 |
| Ibandronate | 13,187 | 3,947 | 2,274 | 1,983 | 1,912 | 1,710 | 1,540 | 1,155 | 1,009 | 900 | 930 | 837 | 7,462 |
| Pamidronate | 30 | <11 | <11 | <11 | <11 | <11 | <11 | <11 | <11 | <11 | <11 | <11 | <11 |
| Raloxifene | 6,182 | 1,797 | 1,181 | 987 | 1,046 | 869 | 904 | 664 | 525 | 578 | 689 | 605 | 9,387 |
| Risedronate | 16,775 | 4,469 | 3,247 | 2,767 | 2,916 | 2,986 | 2,212 | 1,667 | 1,502 | 1,416 | 1,308 | 1,368 | 11,595 |
| Zoledronate | 3,330 | 14 | <11 | <11 | 12 | 125 | 426 | 128 | 55 | 29 | 26 | 53 | 631 |
| Romosozumab | 132 | 51 | 42 | 25 | 16 | 42 | 15 | <11 | <11 | <11 | <11 | <11 | <11 |
| Abaloparatide | 1,419 | 438 | 293 | 236 | 238 | 207 | 173 | 179 | 220 | 128 | 136 | 130 | 53 |
| Teriparatide | 10,518 | 3,537 | 1,921 | 2,756 | 1,712 | 1,798 | 1,181 | 1,123 | 1,209 | 1,360 | 3,163 | 3,394 | 3,437 |

Supplemental Table 6. Osteoporosis treatment pathways demonstrating distribution of 1^st^-, 2^nd^-, 3^rd^-, and 4^th^-line osteoporosis medications prescribed. Cells with <11 patients were censored due to data requirements.

|  | 1^st^ line | 2^nd^ line | 3^rd^ line | 4^th^ line |
| --- | --- | --- | --- | --- |
| Alendronate | 389,772 | 1,366 | 182 | <11 |
| Calcitonin | 37,911 | 450 | 86 | <11 |
| Denosumab | 44,834 | 1,056 | 328 | 37 |
| Etidronate | 136 | <11 | <11 | <11 |
| Ibandronate | 35,639 | 175 | 75 | <11 |
| Pamidronate | 61 | <11 | <11 | <11 |
| Raloxifene | 22,824 | 168 | 89 | <11 |
| Risedronate | 49,824 | 356 | 85 | <11 |
| Zoledronate | 4,522 | 68 | 31 | <11 |
| Abaloparatide | 3,461 | 588 | 98 | <11 |
| Romosozumab | 298 | 67 | 28 | <11 |
| Teriparatide | 27,352 | 3,083 | 313 | <11 |
| Alendronate/Teriparatide | <11 | <11 | <11 | <11 |
| Calcitonin/Teriparatide | <11 | <11 | <11 | <11 |
| Calcitonin/Abaloparatide | <11 | <11 | <11 | <11 |
| Denosumab/Teriparatide | <11 | <11 | <11 | <11 |
| Denosumab/Abaloparatide | <11 | <11 | <11 | <11 |
| Alendronate/Calcitonin | <11 | <11 | <11 | <11 |
| Calcitonin/Denosumab | <11 | <11 | <11 | <11 |
| Calcitonin/Ibandronate | <11 | <11 | <11 | <11 |
| Calcitonin/Raloxifene | <11 | <11 | <11 | <11 |
| Calcitonin/Risedronate | <11 | <11 | <11 | <11 |
| Raloxifene/Abaloparatide | <11 | <11 | <11 | <11 |
| Ibandronate/Raloxifene | <11 | <11 | <11 | <11 |
| Alendronate/Denosumab | <11 | <11 | <11 | <11 |
